# Supplementary material for: Single-cell profiling of trabecular meshwork identifies mitochondrial dysfunction in a glaucoma model that is protected by vitamin B3 treatment
Source: bioRxiv. 2025 Aug 14:2024.11.01.621152. Originally published 2024 Nov 1. Preprint. [Version 2] doi: 10.1101/2024.11.01.621152 (PMC11741249; doi:10.1101/2024.11.01.621152)
Supplement: Supplement 1 [file NIHPP2024.11.01.621152v2-supplement-1.pdf]

bioRxiv preprint doi: https://doi.org/10.1101/2024.11.01.621152; this version posted August 14, 2025. The copyright holder for this preprint (which was not certified by peer review) is the author/funder, who has granted bioRxiv a license to display the preprint in perpetuity. It is made available under aCC-BY 4.0 International license.

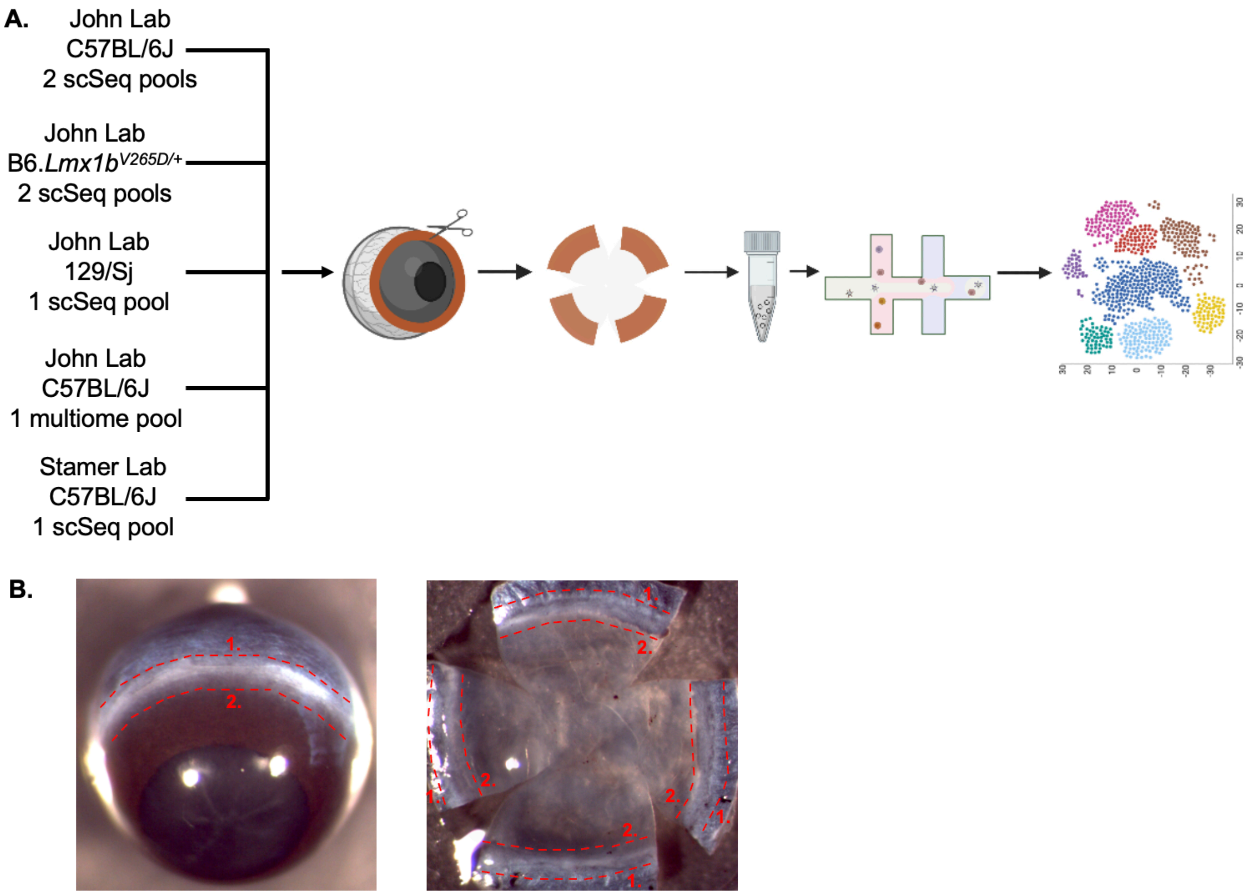


**S1 Fig. Schematic of datasets and pipeline. (A)** Each pool included limbal tissue from 8 eyes. The mouse strains and lab where data were generated are shown (Columbia University, John Lab or Duke, Stamer lab). Individual eyes were dissected to isolate strips of limbal tissue, which are enriched for TM cells in comparison to dissecting the anterior segment as a whole. Limbal strips were then minced and pooled for further processing. Individual cells were sequenced using the 10X Genomics pipeline (see Methods) for sc-RNA-Seq or multiome (snRNA-seq and scATAC-seq). Schematic created with BioRender.com. **(B)** (Left) The limbal region of the enucleated eye is outlined with red dotted lines. The posterior portion of the eye is first removed by making a cut along line1. After lens removal, a second cut is made through the anterior cornea and iris along line 2, generating a limbal strip. This strip is then minced prior to dissociation. (Right) A dissected anterior eye cup (with the lens removed) is shown to indicate the interior surface of the limbus between lines 1 and 2. Centripetal cuts were made in the

bioRxiv preprint doi: https://doi.org/10.1101/2024.11.01.621152; this version posted August 14, 2025. The copyright holder for this preprint (which was not certified by peer review) is the author/funder, who has granted bioRxiv a license to display the preprint in perpetuity. It is made available under aCC-BY 4.0 International license.

cornea of this specimen to flatten it for ease of imaging. Although we do not flatten limbal strips used for RNA-seq, this view provides additional context.

bioRxiv preprint doi: https://doi.org/10.1101/2024.11.01.621152; this version posted August 14, 2025. The copyright holder for this preprint (which was not certified by peer review) is the author/funder, who has granted bioRxiv a license to display the preprint in perpetuity. It is made available under aCC-BY 4.0 International license.

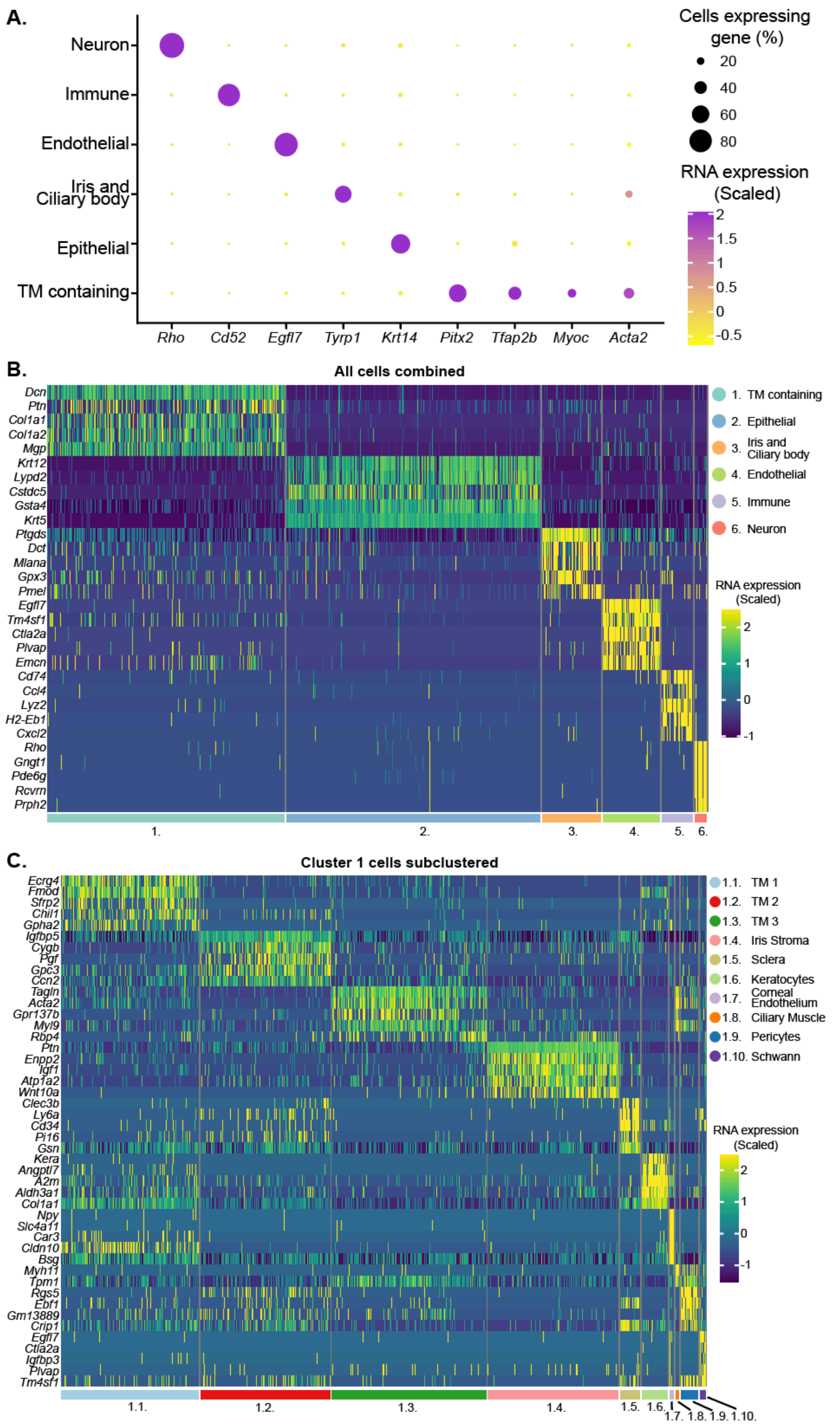

bioRxiv preprint doi: https://doi.org/10.1101/2024.11.01.621152; this version posted August 14, 2025. The copyright holder for this preprint (which was not certified by peer review) is the author/funder, who has granted bioRxiv a license to display the preprint in perpetuity. It is made available under aCC-BY 4.0 International license.

**S2 Fig. Limbal cell cluster marker genes. (A)** The expression levels of various anterior segment cell type marker genes in the integrated B6 and 129 data are depicted on a dot plot. These marker genes are generally accepted to be specific to individual cell types. Epithelial cells (*Krt14*); neurons (*Rho*); endothelial cells (*Egfl7*); ciliary body and iris cells (*Tyrp1*); and immune cells (*Cd52*). Cluster 1 expressed multiple TM marker genes including *Myoc*, *Acta2* (encodes α-SMA), *Pitx2*, and *Tfap2b*. Although some of the neurons may be limbal, it is possible that others are retinal contamination. **(B-C)** Heatmaps of differentially expressed genes across all limbal tissue cell clusters (B) and across the subclusters of cluster 1 (C).

bioRxiv preprint doi: https://doi.org/10.1101/2024.11.01.621152; this version posted August 14, 2025. The copyright holder for this preprint (which was not certified by peer review) is the author/funder, who has granted bioRxiv a license to display the preprint in perpetuity. It is made available under aCC-BY 4.0 International license.

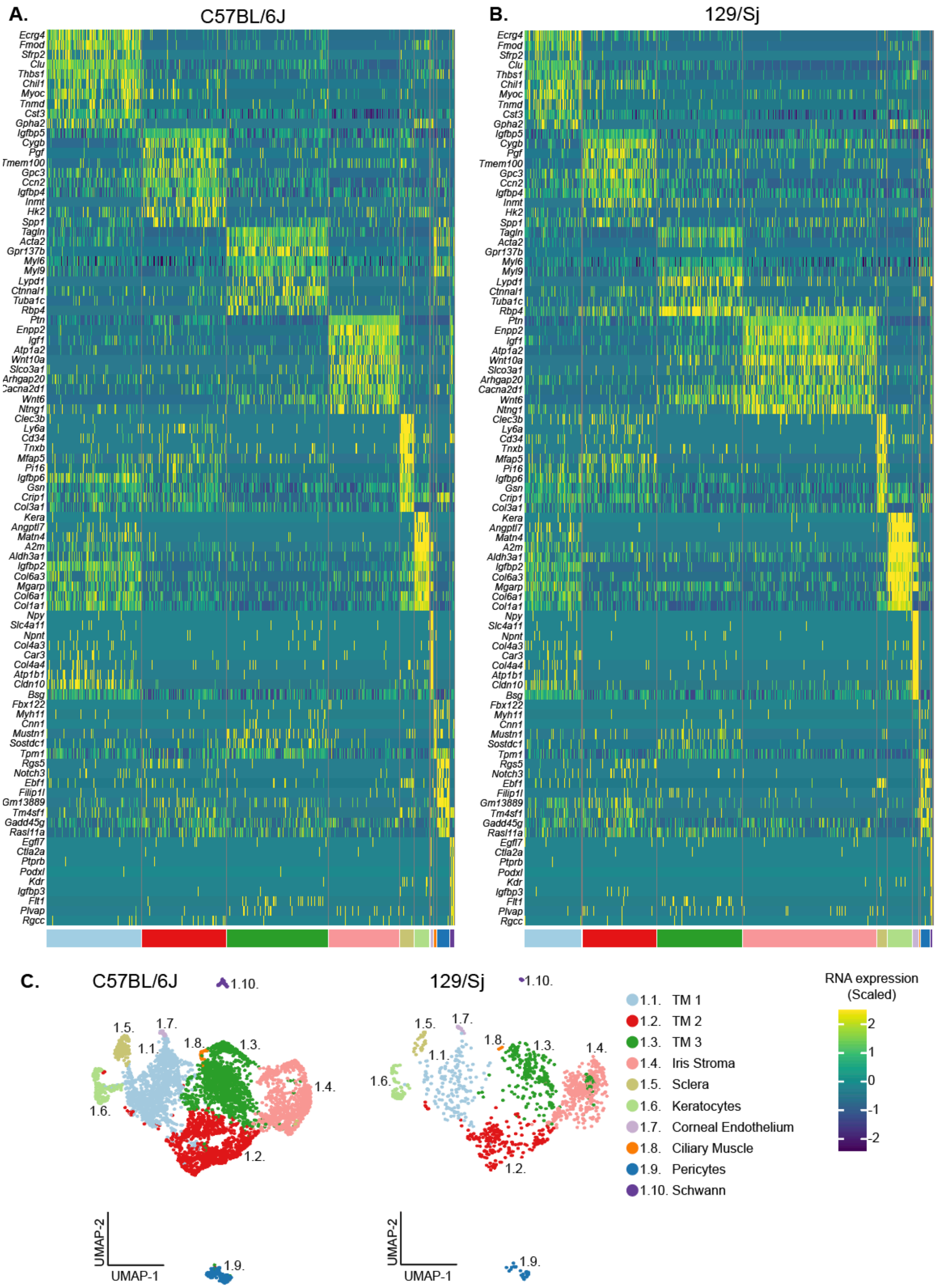


**S3 Fig. Transcriptomic similarities between strain B6 and strain 129.**

**(A-B)** The 129/Sj (129) and C57BL/6J (B6) strains exhibit significant overlap in marker gene expression. Heatmaps display the expression of the top 10 gene markers for each subcluster of

bioRxiv preprint doi: https://doi.org/10.1101/2024.11.01.621152; this version posted August 14, 2025. The copyright holder for this preprint (which was not certified by peer review) is the author/funder, who has granted bioRxiv a license to display the preprint in perpetuity. It is made available under aCC-BY 4.0 International license.

cluster 1. The expression patterns are very similarity across strains. **(C)** UMAP representations of subclusters derived from cluster 1 separated by strain.

bioRxiv preprint doi: https://doi.org/10.1101/2024.11.01.621152; this version posted August 14, 2025. The copyright holder for this preprint (which was not certified by peer review) is the author/funder, who has granted bioRxiv a license to display the preprint in perpetuity. It is made available under aCC-BY 4.0 International license.

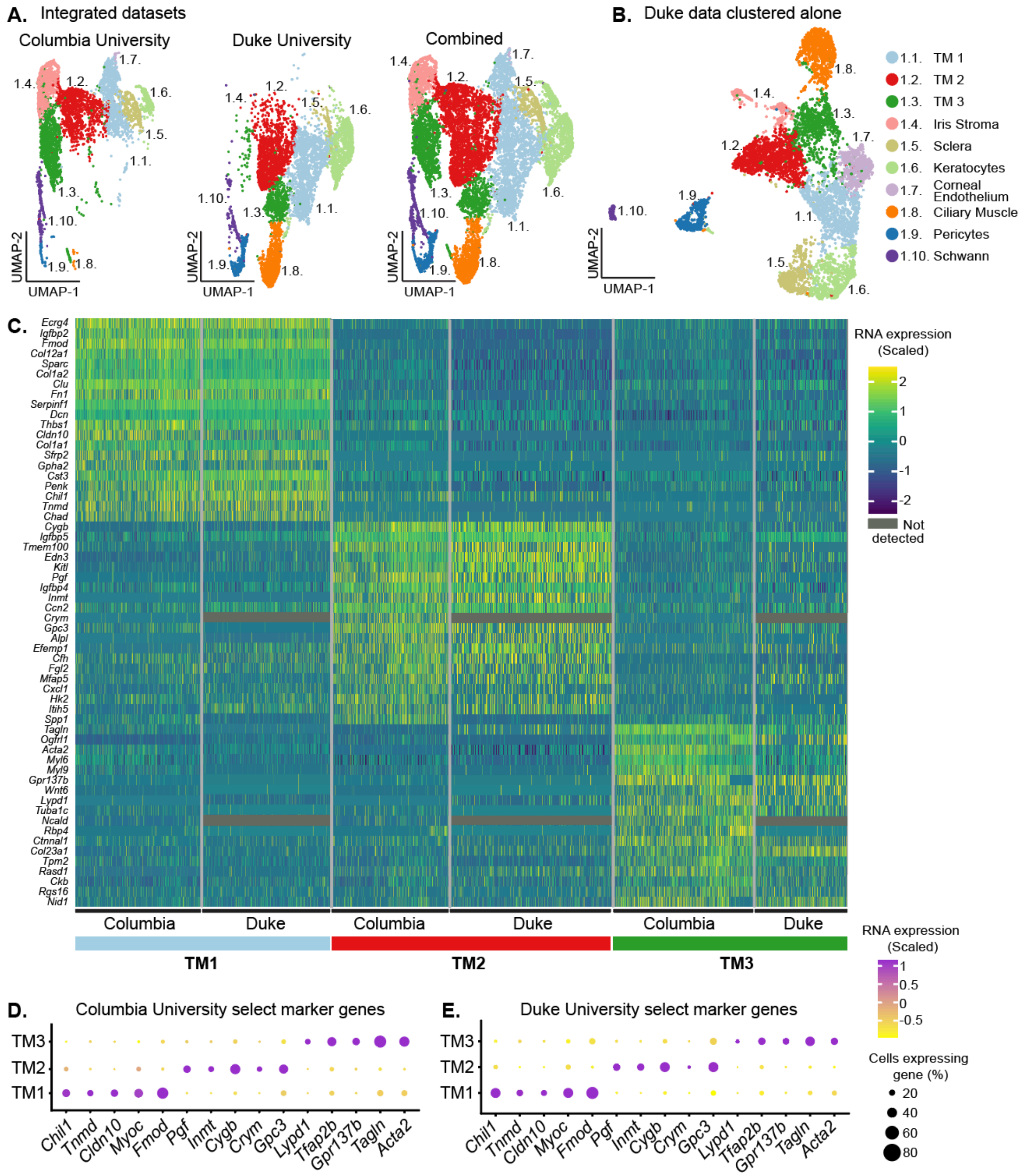


**S4 Fig. An independent B6 scRNA-seq dataset corroborates TM cell clusters. (A)** UMAPs of the integrated Columbia University and Duke University datasets. Cells from the different institutions occupy largely different but adjacent UMAP coordinates with some overlap, indicating batch effects. The difference was largest for TM3, but the marker genes were still

bioRxiv preprint doi: https://doi.org/10.1101/2024.11.01.621152; this version posted August 14, 2025. The copyright holder for this preprint (which was not certified by peer review) is the author/funder, who has granted bioRxiv a license to display the preprint in perpetuity. It is made available under aCC-BY 4.0 International license.

conserved. Because tissue processing techniques can alter gene expression [52], the heatmap variation between institutes likely reflects differences in processing techniques (Methods) and suggests that TM3 cells are more susceptible to these effects than other cell types. **(B)** Analysis of Duke University data alone independently validates our findings presented in *Figure 1* from Columbia University, including the presence of 3 TM cell clusters. **(C)**. A heatmap comparing the top 20 marker genes of each TM cell subcluster for the Columbia (C57BL/6J strain only) and Duke datasets. Overall, there is strong overlap of TM cell gene expression between datasets. Discrepancies include certain marker genes (eg. *Crym*) in the Columbia dataset that are not detected in the Duke dataset. These differences are associated with lower sequencing depth in the Duke data, and other technical factors/batch effects. **(D-E)** Dot plots showing similar TM subtype marker gene expression in the two datasets.

bioRxiv preprint doi: https://doi.org/10.1101/2024.11.01.621152; this version posted August 14, 2025. The copyright holder for this preprint (which was not certified by peer review) is the author/funder, who has granted bioRxiv a license to display the preprint in perpetuity. It is made available under aCC-BY 4.0 International license.

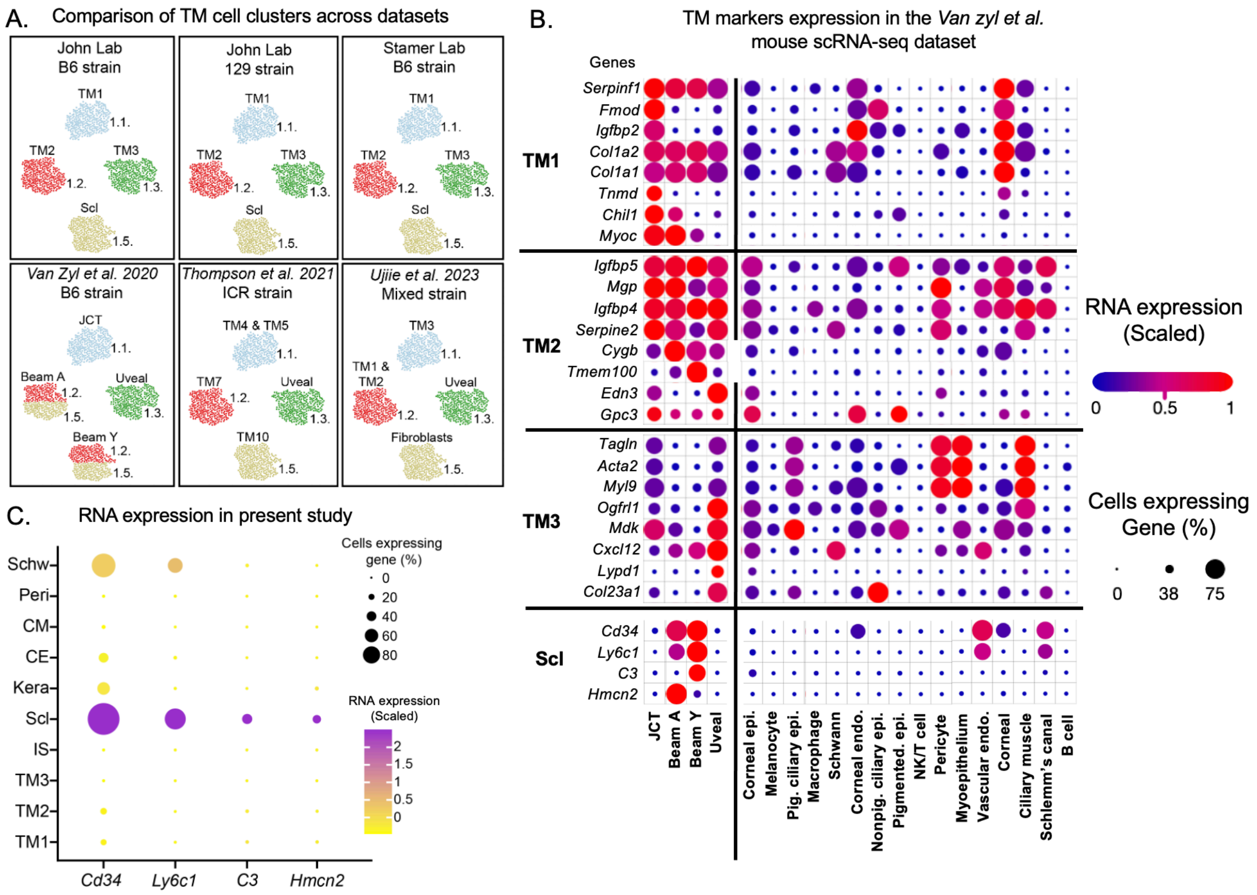


**S5 Fig. Comparison of our TM cells to published mouse datasets (A)** Diagrammatic representation of TM and scleral cell clusters across different mouse scRNA-seq datasets. **Upper panels** diagrammatically represent the current study. **Lower panels** represent the indicated studies. All diagrams based on marker gene expression in the cells that were clustered together. Despite some naming differences, matches for the 3 TM cell types that we identify in the current paper are evident in the previous studies. Although previous studies named additional TM subtypes, we did not subcluster to the same degree to ensure that subtype differences are robust. Previous studies had limited if any validation for some cell types. Based on our current characterization and immunofluorescent localization studies (Figures 2, S6, S8, & S9) some of the cells previously labeled TM beam cells are scleral (possibly fibroblasts). The marker gene expression for these previously named TM beam cells is highest in 'Fibroblasts' rather than TM cells in human scRNA-seq data, further supporting a scleral

bioRxiv preprint doi: https://doi.org/10.1101/2024.11.01.621152; this version posted August 14, 2025. The copyright holder for this preprint (which was not certified by peer review) is the author/funder, who has granted bioRxiv a license to display the preprint in perpetuity. It is made available under aCC-BY 4.0 International license.

identity. In addition, cells previously named as uveal cells are a TM cell type. The cells are color-coded based on their molecular identities to match the current study. Current study subcluster identity numbers are shown. **(B)** Dot plot of TM cell subtype markers identified in the present study (left) using the dataset and cell type assignments (bottom) of Van Zyl et al. Based on these markers, our TM subtypes match their data as follows: TM1= JCT, TM2 = Beam A and Beam Y, and TM3 = Uveal). Scleral markers that we found are enriched in the Van Zyl Beam A and Y cells, which appear to be a mix of different cell types. **(C)** Expression of the Van Zyl Beam A and Y cell markers that are enriched in sclera in our combined B6 and 129 dataset (the same expression pattern was evident in all Columbia and Duke datasets in the current study). See Figure S6 for IF showing expression of these markers in Sclera but not TM.

bioRxiv preprint doi: https://doi.org/10.1101/2024.11.01.621152; this version posted August 14, 2025. The copyright holder for this preprint (which was not certified by peer review) is the author/funder, who has granted bioRxiv a license to display the preprint in perpetuity. It is made available under aCC-BY 4.0 International license.

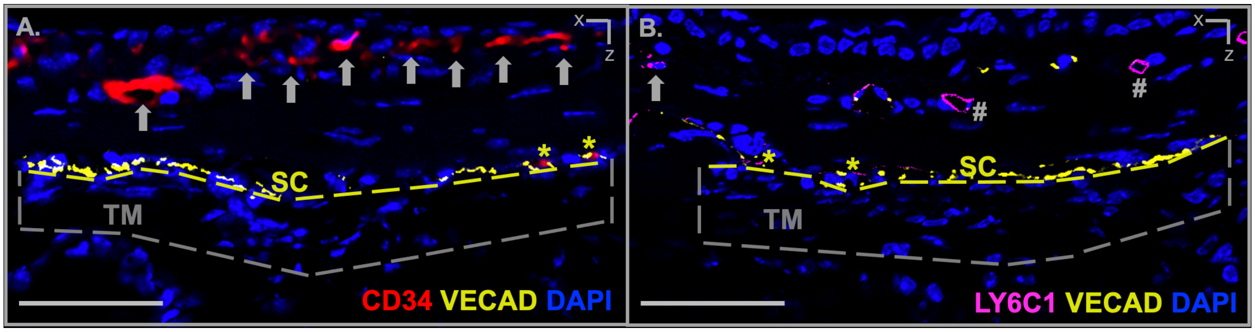


**S6 Fig. Immunostaining demonstrates expression of specific marker genes in sclera but not TM**. (**A**) CD34 is expressed in cells of the sclera/corneoscleral transition zone (arrows). No CD34 staining was found in the TM (> 60 sections from 4 eyes). **(B)** LY6C1 is expressed in both vascular (based on morphology and the vascular endothelial cell marker, VECAD, hash marks) and nonvascular (arrow) cells in the sclera. LY6C1 is not found in the TM. The TM is encompassed within the dashed box while the yellow dashed line marks the inner wall of SC (see Figure S7). CD34 and LY6C1 were expressed in SC endothelial cells (yellow asterisks). Scale bars = 50 μm.

bioRxiv preprint doi: https://doi.org/10.1101/2024.11.01.621152; this version posted August 14, 2025. The copyright holder for this preprint (which was not certified by peer review) is the author/funder, who has granted bioRxiv a license to display the preprint in perpetuity. It is made available under aCC-BY 4.0 International license.

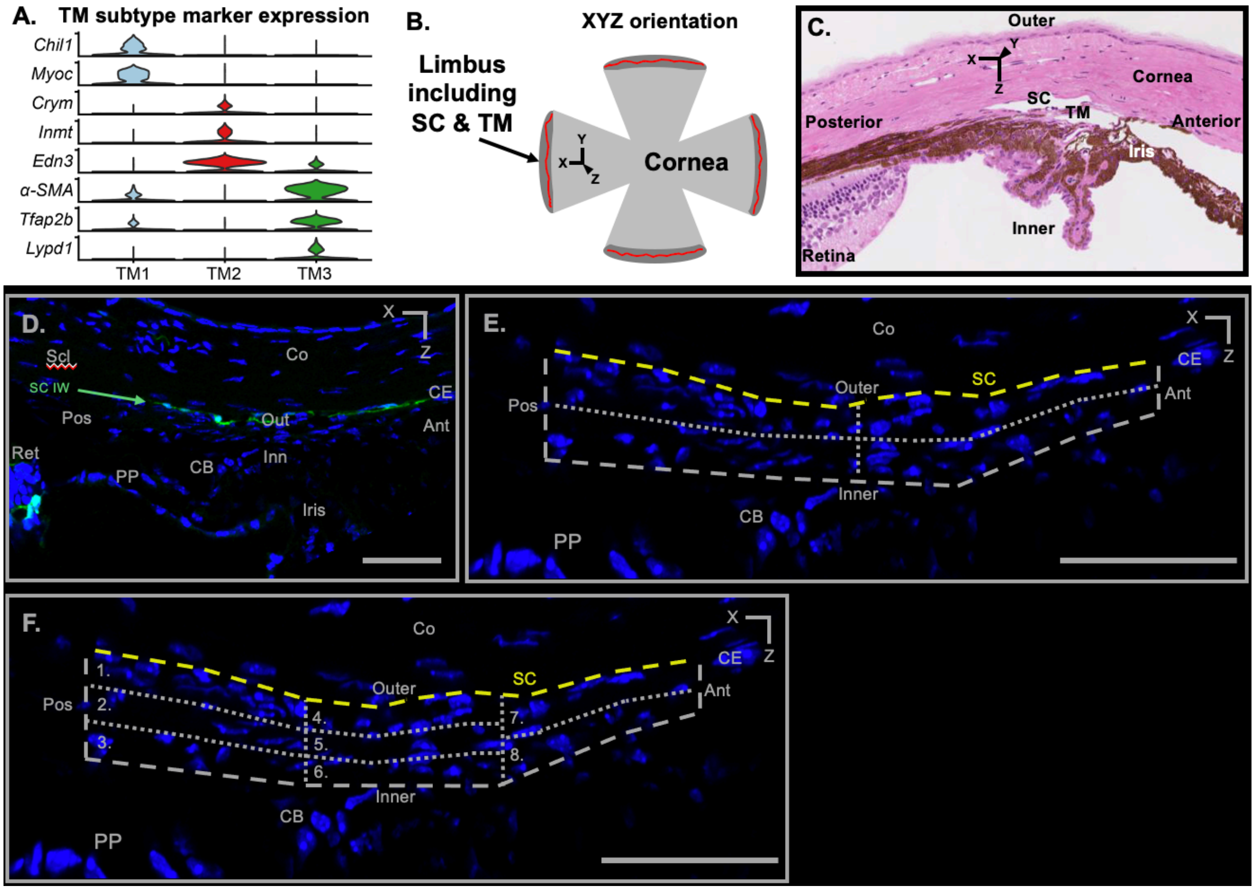


**S7 Fig. Assessing zonal distribution of TM cell subtypes. (A)** Violin plot showing expression of signature genes that were used to localize TM cell subtypes. **(B-C)** Diagram of flat-mounted anterior segment (B) and *hematoxylin and eosin-stained s*agittal section (C) indicating the X, Y and Z axis as used throughout this paper as previously reported (Kizhatil et al., 2014)**. (D-E)** Schlemm's canal (SC) inner wall was identified based on anatomy and the expression of *Prox*-GFP (green) and other endothelial cell markers. The pars plana (PP) and corneal endothelium (CE) were identified based on anatomy and DAPI staining. The posterior boundary of the TM begins just anterior to the PP and was typically aligned with the most posterior portion of SC. The anterior TM ends where the corneal endothelium begins. This typically aligned with the most anterior portion of SC. The outer boundary of the TM was immediately internal to the inner wall of SC. The inner-most TM was where the TM cells end abutting the anterior chamber. Using these boundaries, the anterior-posterior and inner-outer axis of the TM were measured.

bioRxiv preprint doi: https://doi.org/10.1101/2024.11.01.621152; this version posted August 14, 2025. The copyright holder for this preprint (which was not certified by peer review) is the author/funder, who has granted bioRxiv a license to display the preprint in perpetuity. It is made available under aCC-BY 4.0 International license.

Based on these measurements for each eye, the TM was divided in half along both the inner-outer and anterior-posterior axes. (**F**) For more refined analysis, the anterior-posterior distance was then divided into thirds to generate posterior, central, and anterior regions. Each of these regions was then equally divided into their subregions as shown. Because the anterior TM is thinner, it was divided into two zones. These divisions generate 8 TM zones. All scale bars: 50 µm.

bioRxiv preprint doi: https://doi.org/10.1101/2024.11.01.621152; this version posted August 14, 2025. The copyright holder for this preprint (which was not certified by peer review) is the author/funder, who has granted bioRxiv a license to display the preprint in perpetuity. It is made available under aCC-BY 4.0 International license.

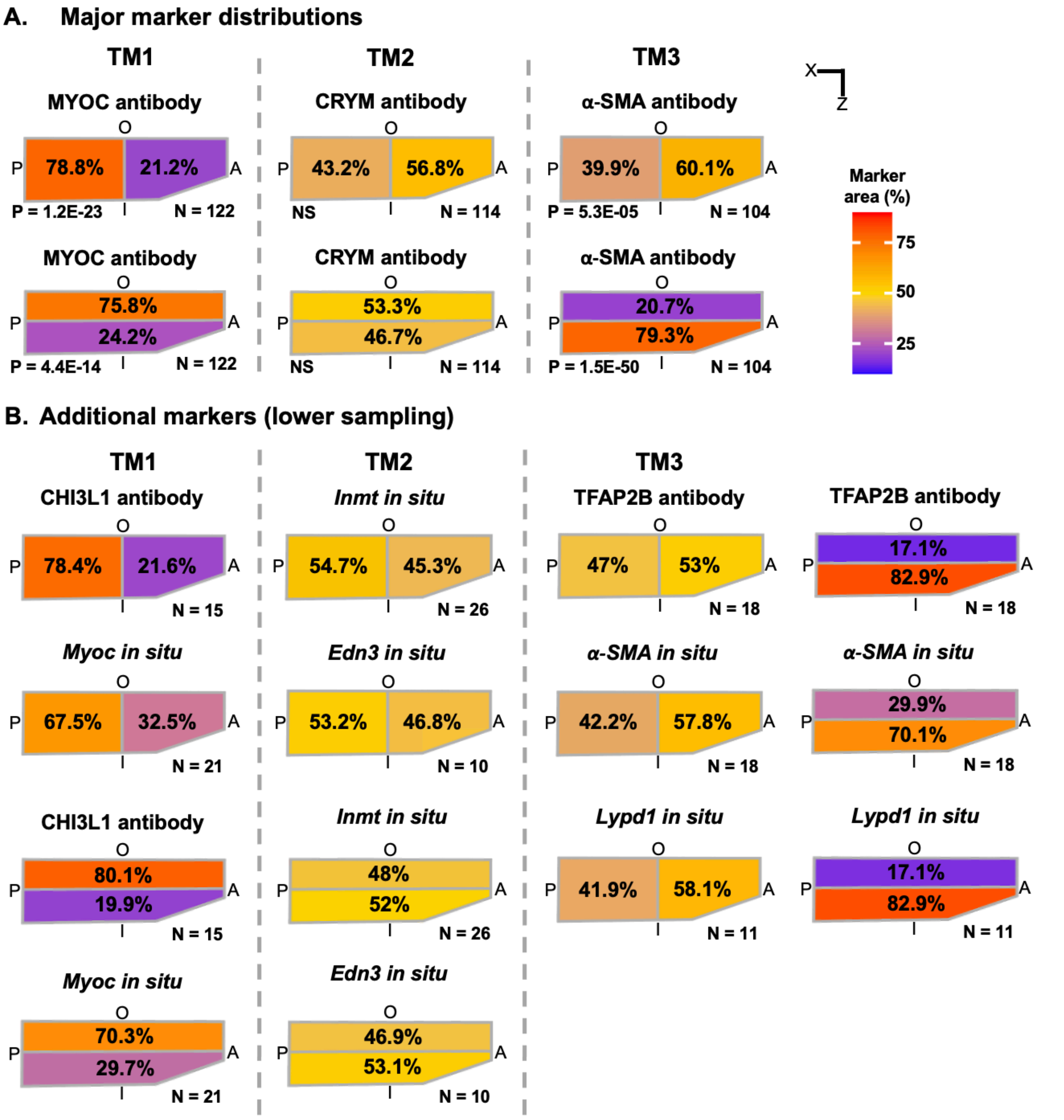


**S8 Fig. Distribution of TM cell subtypes by marker. (A)** Schematic representation of major marker distributions from Figure 2. **(B)** Additional markers for a given TM cell subtype had similar patterns of localization (see Figure S9 for example staining). However, these markers were not sampled deeply enough to perform statistical analysis. A: anterior TM, I: inner TM, O: outer TM, P: posterior TM.

bioRxiv preprint doi: https://doi.org/10.1101/2024.11.01.621152; this version posted August 14, 2025. The copyright holder for this preprint (which was not certified by peer review) is the author/funder, who has granted bioRxiv a license to display the preprint in perpetuity. It is made available under aCC-BY 4.0 International license.

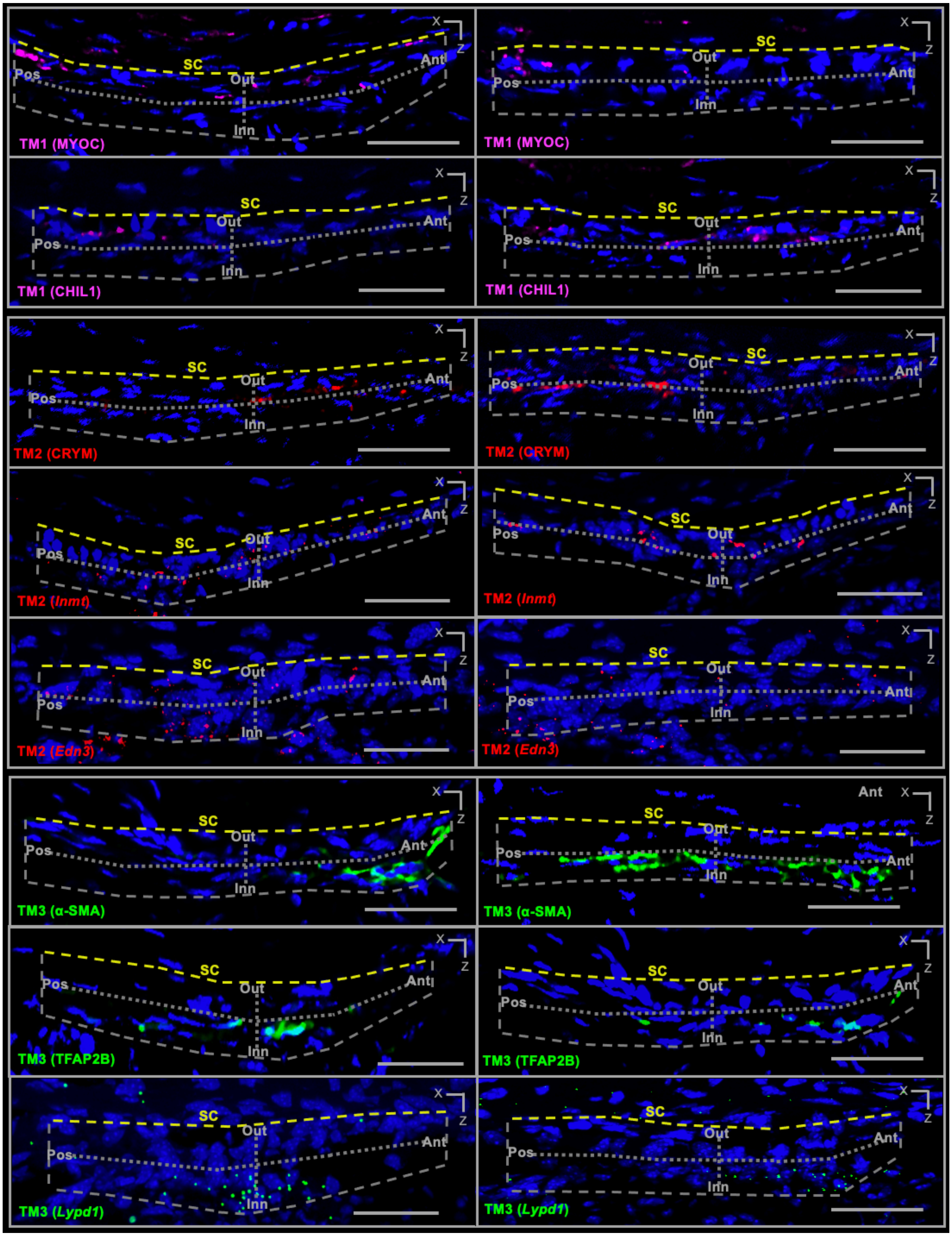

bioRxiv preprint doi: https://doi.org/10.1101/2024.11.01.621152; this version posted August 14, 2025. The copyright holder for this preprint (which was not certified by peer review) is the author/funder, who has granted bioRxiv a license to display the preprint in perpetuity. It is made available under aCC-BY 4.0 International license.

**S9 Fig. Additional examples of TM subtype marker localization.** Although there is variability in expression between sections, the aggregate of all sections reveals the biased distributions of TM cell subtypes as summarized in Figures 2. Here we focus on TM expression. However, some of the markers are also expressed outside of the TM. For examples, *Edn3* is expressed in vasculature, while both *Lypd1* and *Inmt* are expressed at low levels in scleral and iris cells (but are higher in TM cells). All scale bars: 50 μm. Markers assessed by IF: MYOC, CHIL1, CRYM, α-SMA, TFAP2B. Markers assessed by ISH: *Inmt*, *Edn3*, *Lypd1*.  All scale bars: 50 μm.

bioRxiv preprint doi: https://doi.org/10.1101/2024.11.01.621152; this version posted August 14, 2025. The copyright holder for this preprint (which was not certified by peer review) is the author/funder, who has granted bioRxiv a license to display the preprint in perpetuity. It is made available under aCC-BY 4.0 International license.

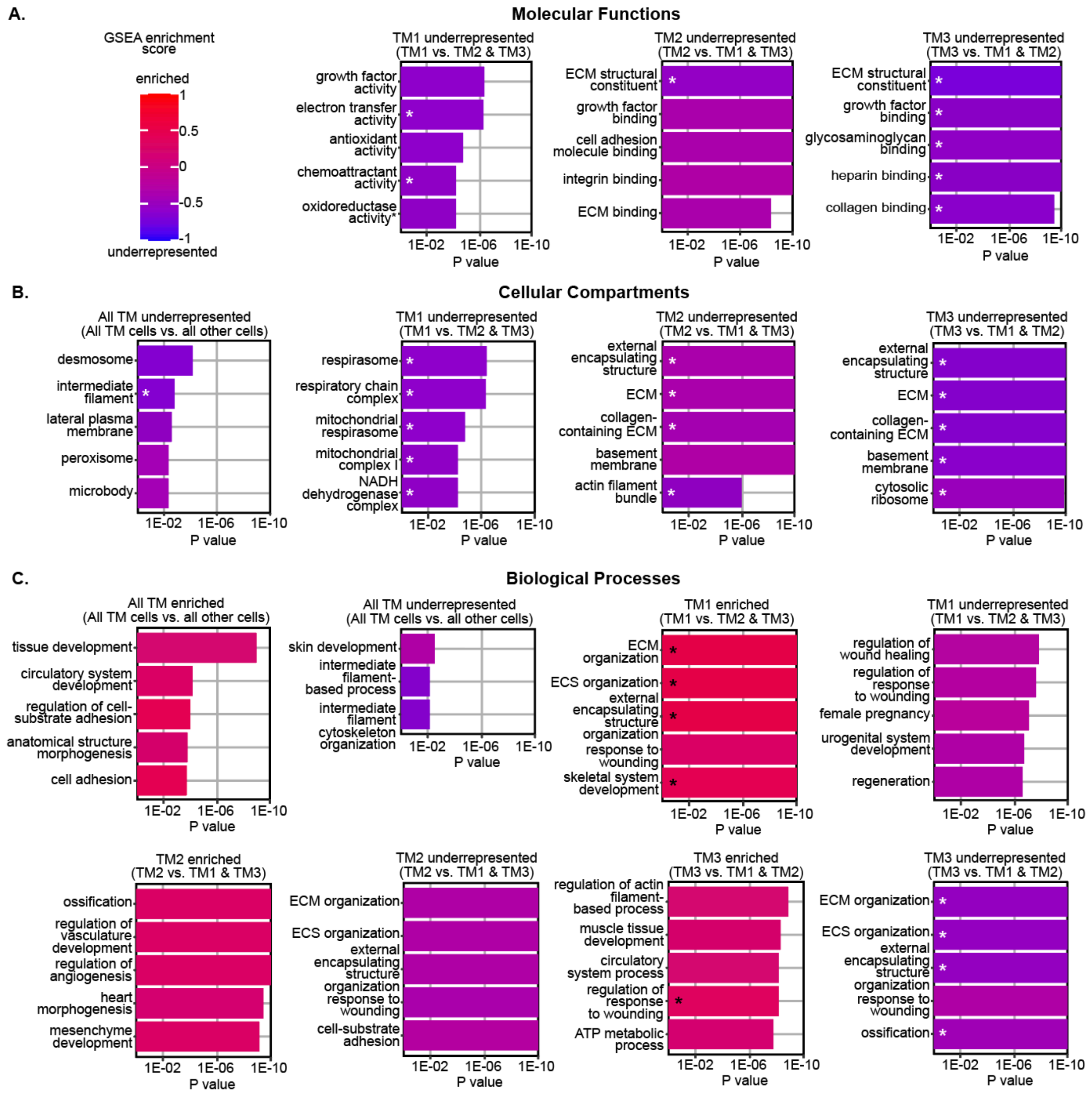


**S10 Fig. TM cell pathway analysis. (A-C)** Additional analyses showing the most significant pathways (by gene ontology (GO) analysis. Pathways are further separated into enriched or underrepresented categories based on gene set enrichment analysis (GSEA) score. Asterisks indicate pathways that are significantly enriched or underrepresented based on GSEA score. ECM = extracellular matrix. ECS = extracellular structure.

bioRxiv preprint doi: https://doi.org/10.1101/2024.11.01.621152; this version posted August 14, 2025. The copyright holder for this preprint (which was not certified by peer review) is the author/funder, who has granted bioRxiv a license to display the preprint in perpetuity. It is made available under aCC-BY 4.0 International license.

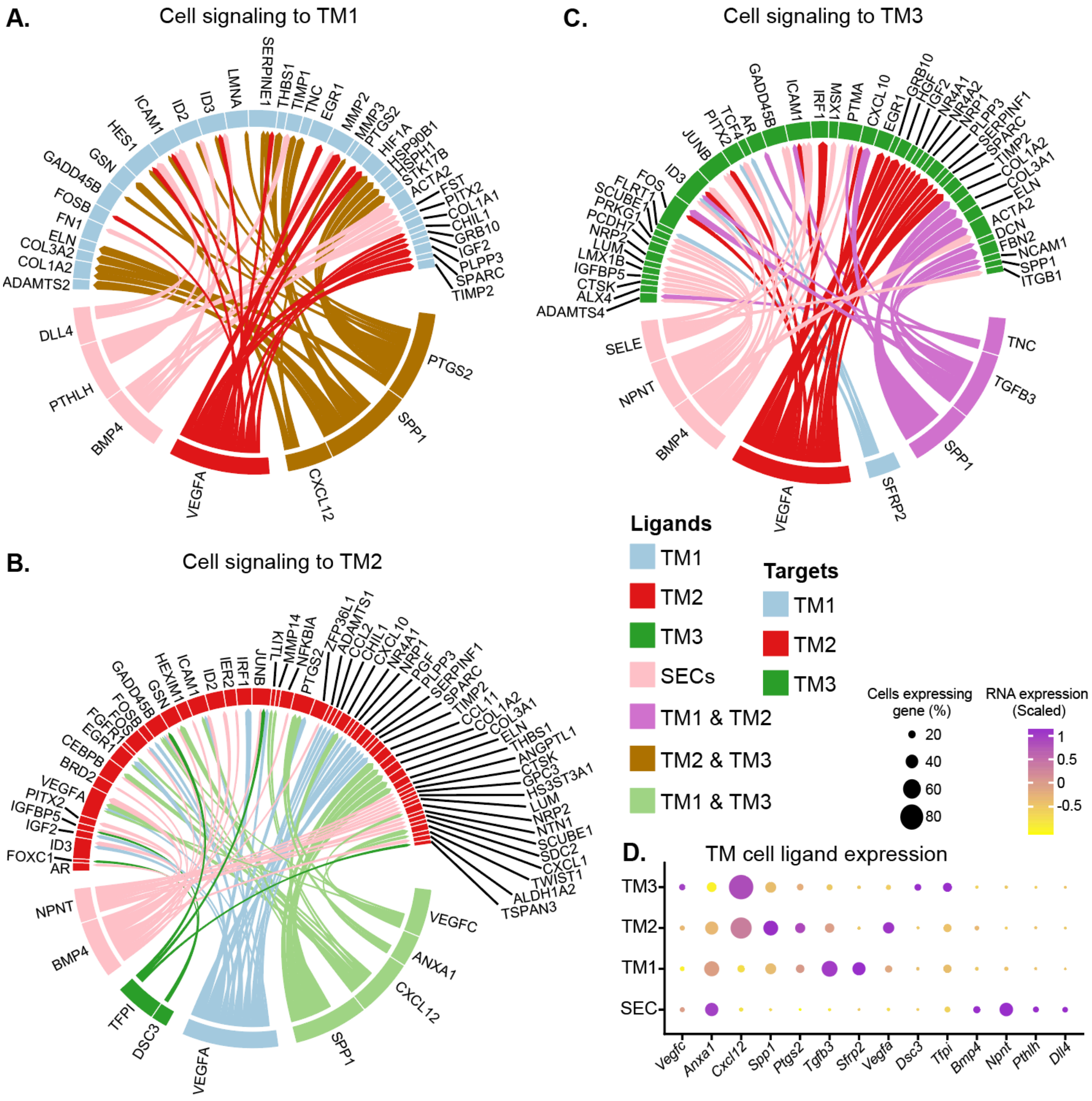


**S11 Fig. TM-TM and SEC-TM signaling. (A-C)** Analyses showing the top predicted interactions between TM and Schlemm's canal endothelial (SEC) ligands and individual TM cell target molecules (Circos plots). The three plots show target molecules in each individual TM cell subtype respectively. **(D)** The expression of all ligands predicted to have signaling interactions with TM cell subtypes (Dot plot).

bioRxiv preprint doi: https://doi.org/10.1101/2024.11.01.621152; this version posted August 14, 2025. The copyright holder for this preprint (which was not certified by peer review) is the author/funder, who has granted bioRxiv a license to display the preprint in perpetuity. It is made available under aCC-BY 4.0 International license.

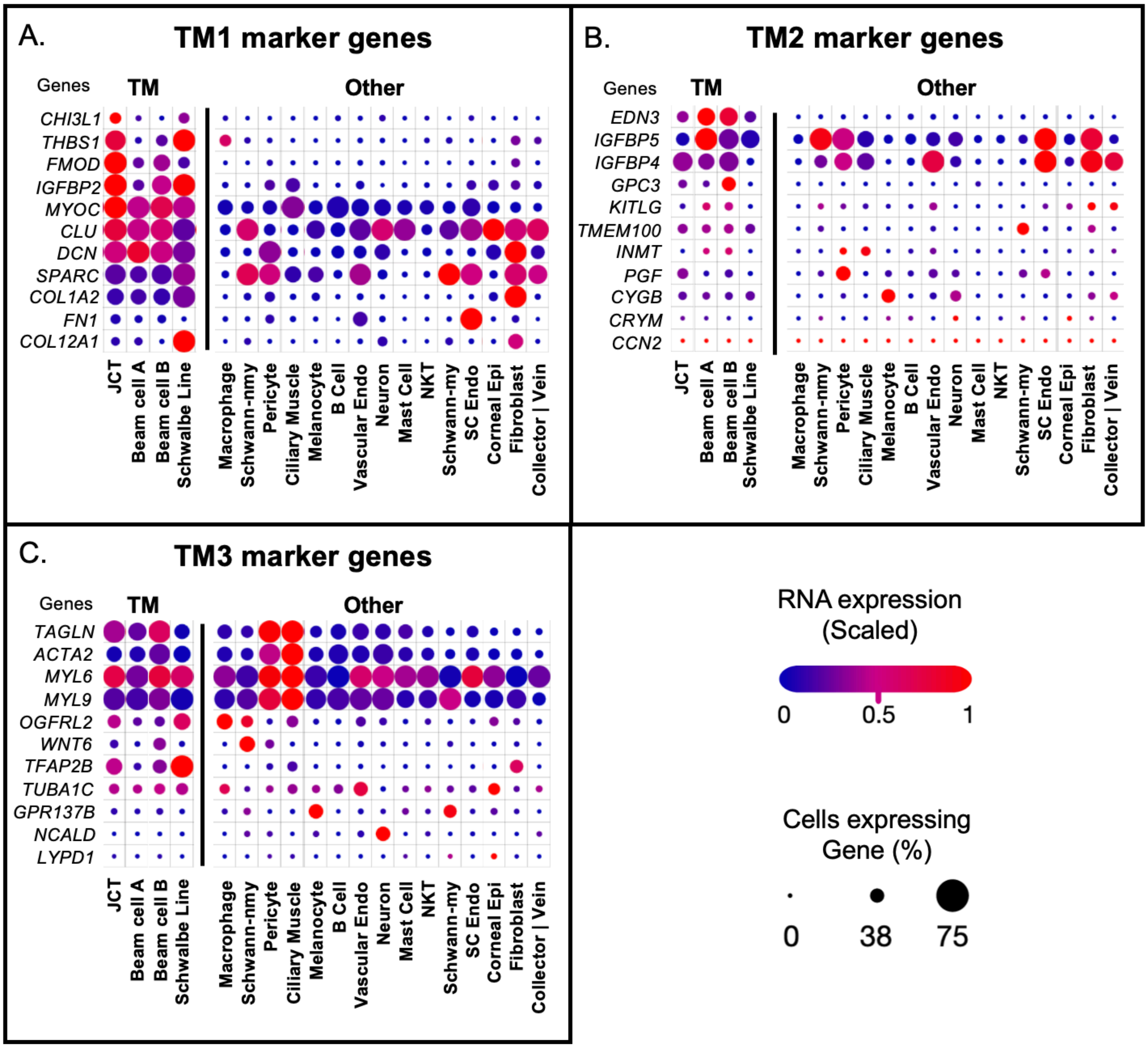


**S12 Fig. Mouse TM subtype marker expression in human TM cells. (A-C)** Dot plots of the expression of the top 11 marker genes for each of our mouse TM cell subtypes that have human orthologues expressed in the Van Zyl et al human dataset. TM1 marker genes generally have higher expression in the human JCT cluster than in any other cluster of cells. Other than EDN3, TM2 and TM3 marker genes do not show a strong enrichment for the annotated TM cells compared to other cells. Among TM cells, TM2 marker genes are more enriched in beam A and beam B cells. TM3 markers are mostly expressed in relatively equal levels across all the human TM cells. Schwalbe's line cells are also similar to TM. The anterior insertion of the TM is near

bioRxiv preprint doi: https://doi.org/10.1101/2024.11.01.621152; this version posted August 14, 2025. The copyright holder for this preprint (which was not certified by peer review) is the author/funder, who has granted bioRxiv a license to display the preprint in perpetuity. It is made available under aCC-BY 4.0 International license.

Schwalbe’s line, where TM stem cells are reported to reside. Therefore, the Schwalbe’s line cells maybe TM stem cells.

bioRxiv preprint doi: https://doi.org/10.1101/2024.11.01.621152; this version posted August 14, 2025. The copyright holder for this preprint (which was not certified by peer review) is the author/funder, who has granted bioRxiv a license to display the preprint in perpetuity. It is made available under aCC-BY 4.0 International license.

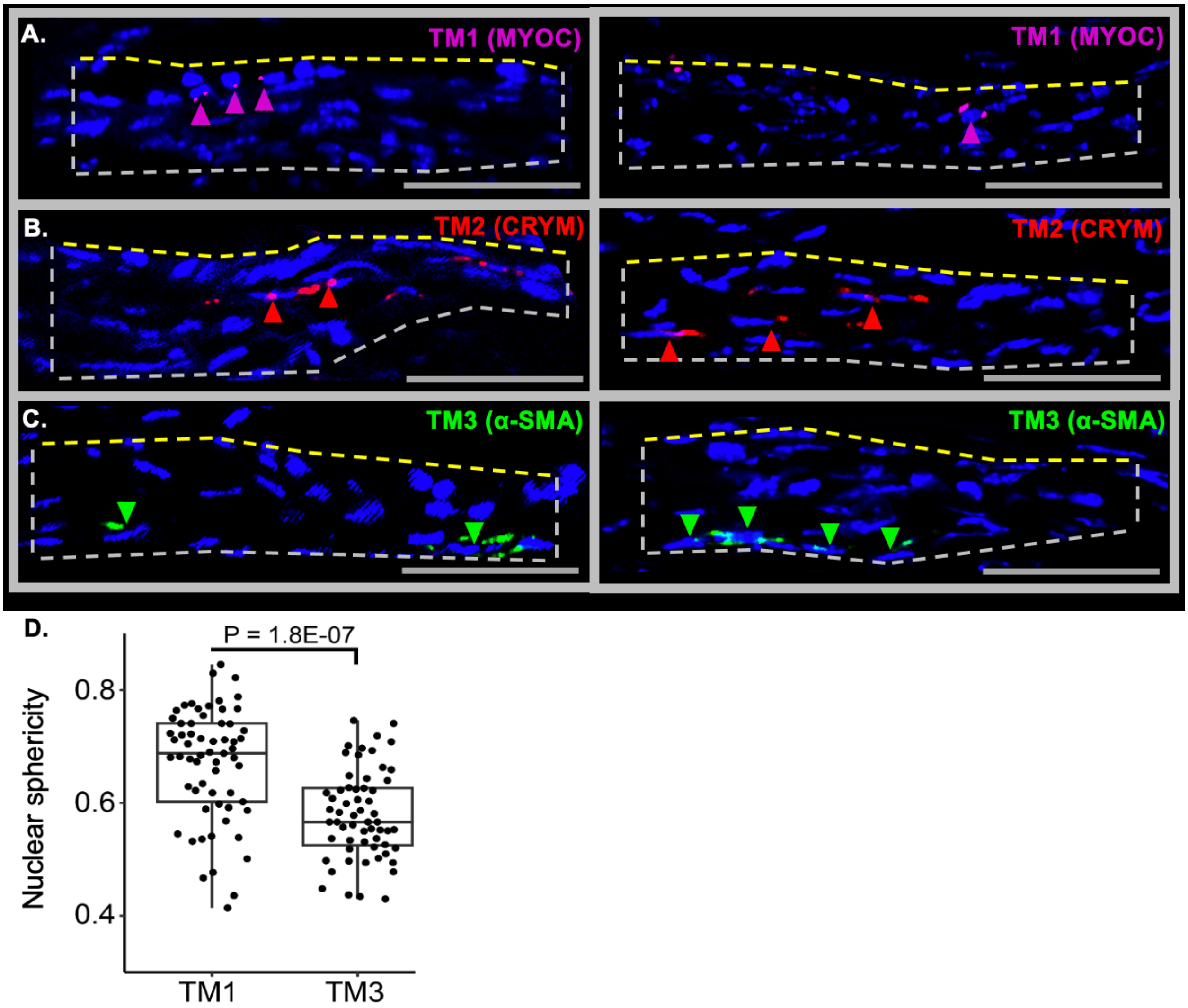


**S13 Fig. Nuclear morphology of each TM subtype. (A-C)** 2D section view of TM cell subtype nuclei. TM1 nuclei adjacent to the SC appear shorter than TM2 and TM3 nuclei and lack the typical elongated, endothelial-like morphology of beam cells. This shorter morphology gives TM1 nuclei a more spherical appearance. All scale bars: 50 μm. **(D)** To rigorously assess whether TM1 nuclei are more spherical, we analyzed their reconstructed 3D shapes from whole mounts images by confocal microscopy, comparing them to TM3 nuclei using the 'Sphericity' tool in Imaris. The data show that TM1 cells indeed have more spherical nuclei, consistent with a JCT identity. However, some TM1 cells exhibit more elongated nuclei, especially when located further from the SC. A higher sphericity score indicates a more rounded nucleus (with a

bioRxiv preprint doi: https://doi.org/10.1101/2024.11.01.621152; this version posted August 14, 2025. The copyright holder for this preprint (which was not certified by peer review) is the author/funder, who has granted bioRxiv a license to display the preprint in perpetuity. It is made available under aCC-BY 4.0 International license.

completely spherical shape scoring 1). We characterized 60 randomly selected nuclei for each TM cell subtype. Groups were compared using Student's t-test.

bioRxiv preprint doi: https://doi.org/10.1101/2024.11.01.621152; this version posted August 14, 2025. The copyright holder for this preprint (which was not certified by peer review) is the author/funder, who has granted bioRxiv a license to display the preprint in perpetuity. It is made available under aCC-BY 4.0 International license.

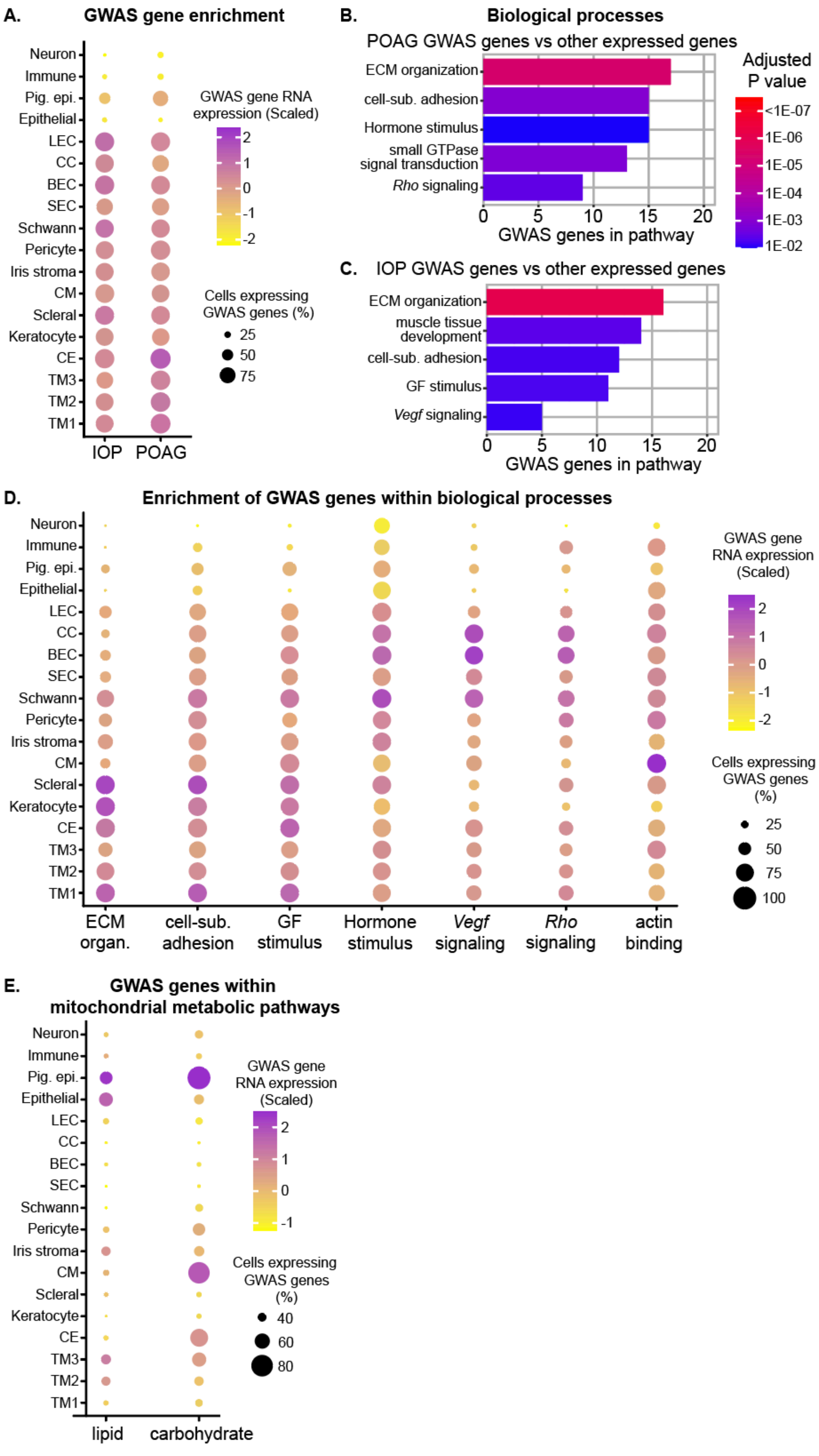


599

bioRxiv preprint doi: https://doi.org/10.1101/2024.11.01.621152; this version posted August 14, 2025. The copyright holder for this preprint (which was not certified by peer review) is the author/funder, who has granted bioRxiv a license to display the preprint in perpetuity. It is made available under aCC-BY 4.0 International license.

**S14 Fig: GWAS gene expression in limbal cells. (A)** Expression of genes implicated in risk for IOP elevation (left) and primary open-angle glaucoma (POAG, right) in humans GWAS plotted against mouse limbal cell type. There is similar GWAS gene expression across TM cell subtypes and other limbal cell types. TM1 and TM2 have a slightly higher expression of GWAS genes compared to TM3. **(B-C)** Pathway analysis (Gene ontology, GO) shows enrichment of biological pathways for the POAG (B) or IOP (C) genes vs other expressed genes considering all cell types (see Table S5 for full list of GWAS gene-enriched pathways). **(D)** Enriched biological pathways among POAG GWAS genes within the indicated cell types. **(E)** Cell type enrichment of lipid and carbohydrate metabolism pathway genes that are significantly associated with POAG (Khawaja et al.). ECM organ. = extracellular matrix organization, cell-sub. = cell-substrate adhesion, Hormone stimulus = cellular response to hormone stimulus, small GTPase signal transduction = regulation of small GTPase mediated signal transduction, *Rho* signaling = regulation of Rho protein signal transduction, GF stimulus = regulation of cellular response to growth factor stimulus, *Vegf* signaling = vascular endothelial growth factor receptor signaling pathway.

bioRxiv preprint doi: https://doi.org/10.1101/2024.11.01.621152; this version posted August 14, 2025. The copyright holder for this preprint (which was not certified by peer review) is the author/funder, who has granted bioRxiv a license to display the preprint in perpetuity. It is made available under aCC-BY 4.0 International license.

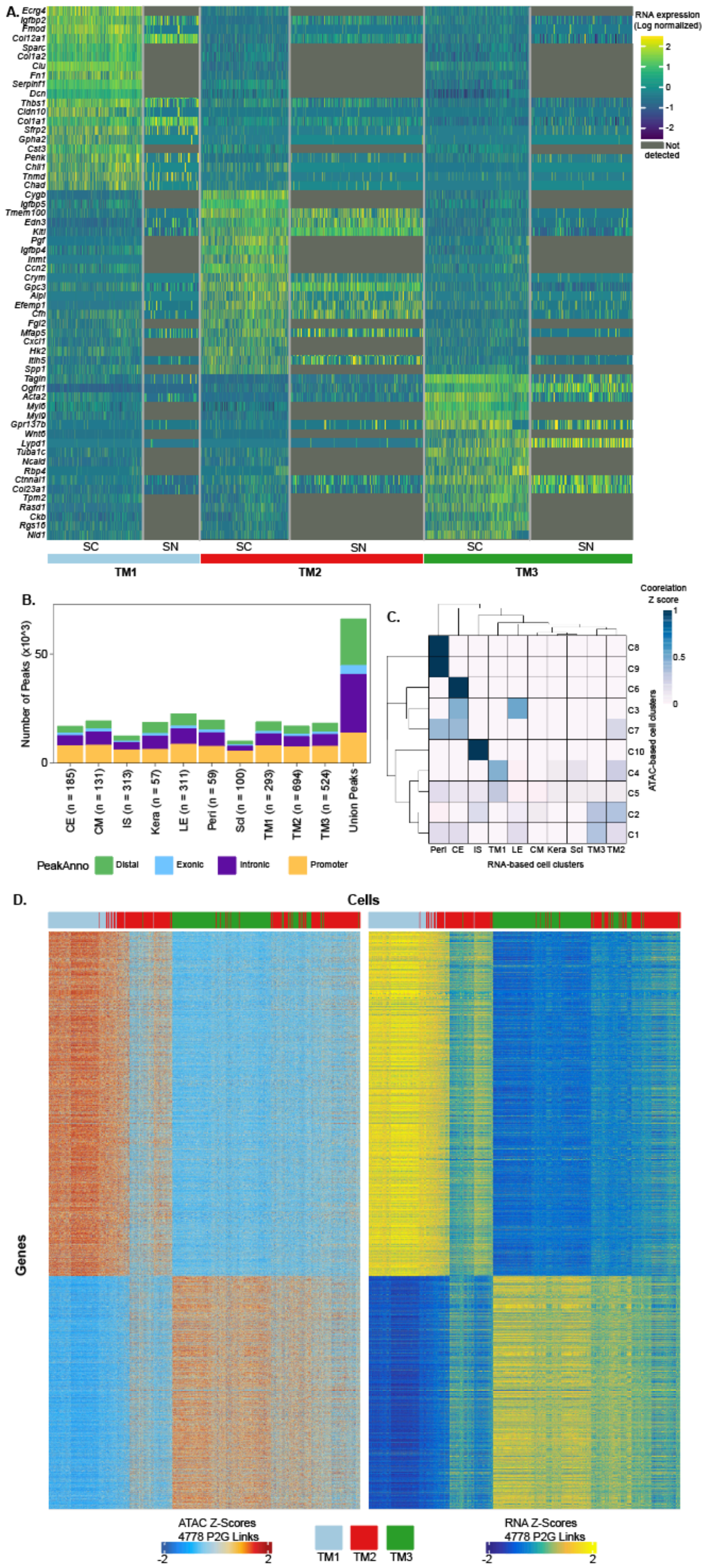

bioRxiv preprint doi: https://doi.org/10.1101/2024.11.01.621152; this version posted August 14, 2025. The copyright holder for this preprint (which was not certified by peer review) is the author/funder, who has granted bioRxiv a license to display the preprint in perpetuity. It is made available under aCC-BY 4.0 International license.

**S15 Fig. Analysis of ATAC dataset (A)** Overlap of marker gene expression for each TM cell subtype between single-cell (sc) and single-nucleus (sn) RNA sequencing (RNA-seq). Other than the expected technical drop out of genes (no detected expression) in the snRNA-seq dataset, there is general agreement. **(B)** Localization of significantly open chromatin regions (snATAC-seq). **(C)** Confusion matrix of clusters identified by snRNA-seq and snATAC-seq after separately clustering each data type using unbiased dimensional reduction. Colors indicate the fraction of cells identified in each ATAC cluster (row) which are also identified in each RNA cell type (columns), where darker colors represent stronger correspondence between RNA and ATAC clusters. There is a significant correlation between gene expression (RNA-based clustering) and chromatin accessibility (ATAC-based clustering) with adjusted Rand index of 0.20 ($p < 0.001$, permutation test). **(D)** Heatmaps comparing the open chromatin score at a gene promoter (left panel, snATAC-seq) to the RNA expression (right panel, snRNA-seq). Individual genes are represented on the y-axis and individual cells are plotted on the x-axis (only TM cells included). The consistent heatmap patterns indicate a strong overlap between promoter chromatin states and RNA expression for individual genes across cell types, validating the quality of these multiome datasets.

bioRxiv preprint doi: https://doi.org/10.1101/2024.11.01.621152; this version posted August 14, 2025. The copyright holder for this preprint (which was not certified by peer review) is the author/funder, who has granted bioRxiv a license to display the preprint in perpetuity. It is made available under aCC-BY 4.0 International license.

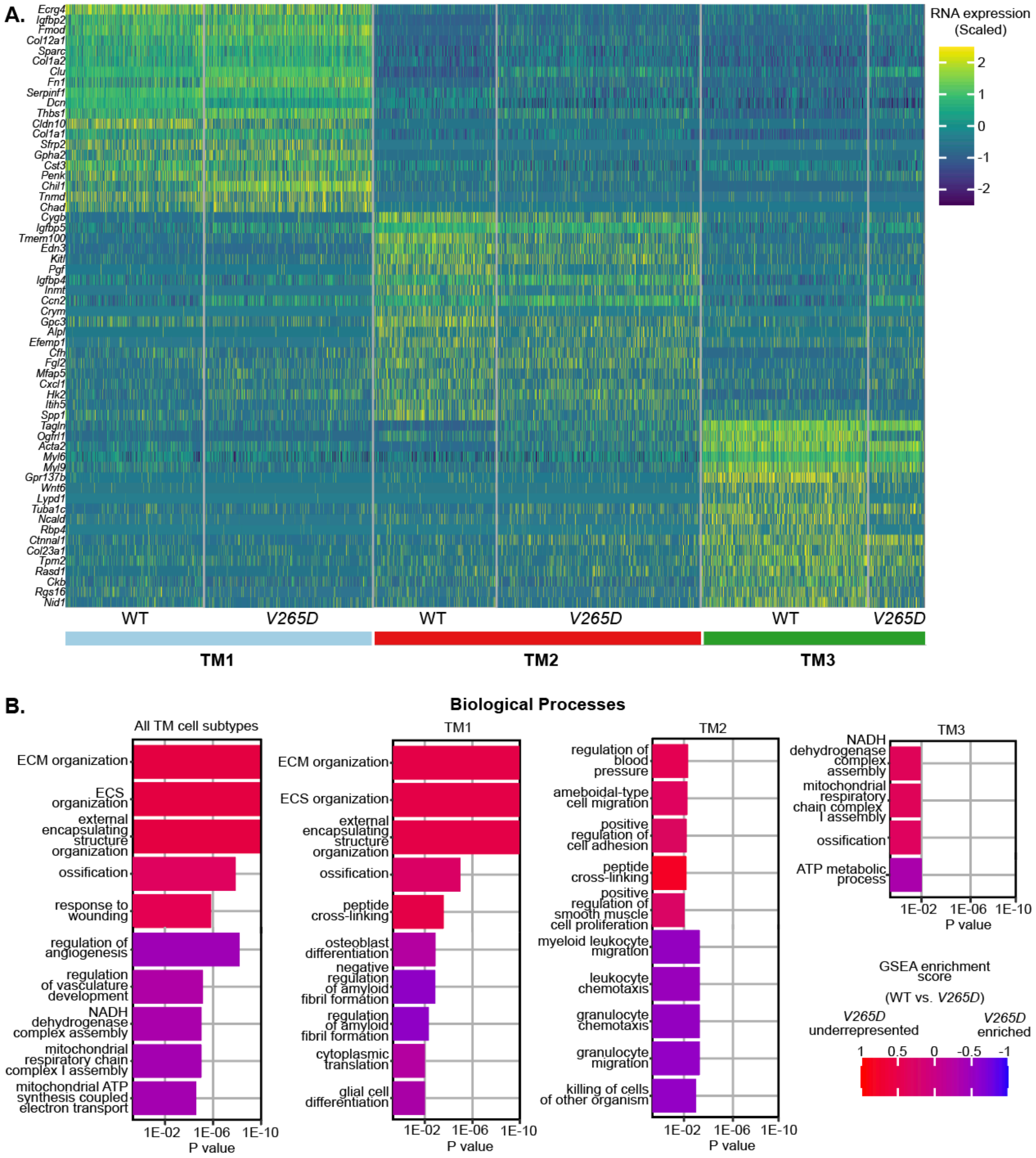


**S16 Fig. Further pathway analyses of *Lmx1b*$^{V265D}$ vs WT. (A)** Heatmaps comparing marker genes for all TM cell subtypes across genotype. **(B)** The top 10 *V265D* upregulated and downregulated biological processes for the indicated comparisons across genotype are shown (all scRNA-seq data, Adjusted P values, axis cut of at 1E-10). ECM = extracellular matrix. ECS = extracellular structure.
